# Adhering to COVID-19 health guidelines: A behavioral-failure perspective

**DOI:** 10.3389/fpsyg.2022.916960

**Published:** 2022-08-02

**Authors:** Zohar Rusou, Irene Diamant

**Affiliations:** ^1^Department of Psychology, The Open University of Israel, Ra'anana, Israel; ^2^School of Behavioral Sciences, The Academic College of Tel Aviv-Yafo, Tel Aviv-Yafo, Israel

**Keywords:** covid-19, failure proneness, human error, guidelines adherence, health adherence, Conscientiousness

## Abstract

The mitigation of pandemics like that caused by the current COVID-19 virus is largely dependent on voluntary public adherence to government rules and regulations. Recent research has identified various individual covariates that account for some of the variance in compliance with COVID-19 behavioral guidelines. However, despite considerable research, our understanding of how and why these factors are related to adherence behavior is limited. Additionally, it is less clear whether disease-transmitting behaviors during a pandemic can be understood in terms of more general behavioral tendencies. The current research has examined the utility of a behavioral-failure lens in predicting adherence to COVID-19 guidelines and in illuminating mechanisms underlying the previously established relationship between Conscientiousness and adherence. In the two studies reported here, individual variations in the predisposition to behavioral failures predicted adherence to COVID-19 measures, and mediated the relationships between Conscientiousness and adherence. The Failure Proneness (FP) questionnaire predicted compliance with COVID-19 guidelines, while the Cognitive Failure Questionnaire (CFQ) did not. The results of hierarchical regressions showed that COVID-19 behavior was predicted only through the intentional factors (and mainly by Noncompliance-Violations). Hence, our data lend support to the notion that noncompliance with official COVID-19 prevention guidelines is driven mainly by intentional factors related to violation of norms and rules. The theoretical and practical implications of this finding are discussed.

## Introduction

The coronavirus pandemic (COVID-19) has posed an exceptional health emergency situation, with apparent economic and emotional consequences. There is currently a lack of consensus on precisely what the coming decade might look like. Some Researchers and health practitioners state that COVID-19 will be controlled “like flu” with “seasonal vaccines,” while others state that even with large-scale use of vaccines, the risk of a sustained spread of new virus mutations will remain for the foreseeable future, and that some mutations could evade vaccine immunity and be more contagious and lethal than current mutations (Kupferschmidt, 2021; Rubin, 2021; Oliu-Barton et al., 2022). Moreover, mounting anthropogenic environmental changes, coupled with a globalized network of travel and trade, allow zoonotic pathogens such as corona-viruses to spill over into human beings with increasing frequency, making us supremely susceptible to their international spread (Castillo-Chavez et al., 2015). Much of the research effort across the past 2 years has involved the necessity of “building the airplane as we fly.” However, in order to support planning for the next pandemic challenge, we should take the opportunity to continue the research and carefully plan future strategies aimed at the containment of the spread (Altmann and Boyton, 2022).

There is now substantial evidence that human behavior plays a fundamental role in the propagation of the COVID-19 virus. A large-scale adoption of officially recommended measures (e.g., social distancing and personal hygiene) has been a key factor for reducing the spread of the pandemic and preventing recurring waves (Alagoz et al., 2020; Cowling et al., 2020; Islam et al., 2020; Mahase, 2020). Nevertheless, unsatisfactory compliance with these measures poses a major challenge in arresting the transmission of the virus (Clark et al., 2020; Mervosh et al., 2020; Wong et al., 2020). Previous research has shown that individual differences in various demographic, personality, attitudinal, and even cognitive factors predict compliance with the official prevention guidelines (Bogg and Milad, 2020; Bailey et al., 2021). However, despite considerable research, our understanding of how and why these factors are related to adherence behavior is limited. Another open question concerns the extent to which disease-transmitting behaviors during a pandemic can be understood in terms of more general behavioral tendencies. We suggest that given the grave implications of COVID-19, noncompliance with prevention guidelines should be regarded as a form of unsafe behavior that elevates the risk for accidents and mishaps (the propagation of the disease). Despite the similarities between noncompliance to the prevention measure and unsafe behavior these topics have largely been discussed in separate literatures. Thus, we believe a typology of behavioral-failure which is employed in the safety literature could prove useful in identifying distinct routes to noncompliance, and illuminate pathways through which individuals’ characteristics are associated with adherence behavior. We suggest that a meaningful consideration of individual differences in adherence to COVID-19 prevention guidelines requires a multifaceted perspective, incorporating various categories of both deliberate and unintended behaviors. The current research addressed the issue through a multifaceted behavioral-failure lens, and focused on Conscientiousness as a test case.

### Individual differences in compliance with COVID-19 guidelines

Recent research has identified various individual covariates that account for some of the variance in compliance with COVID-19 behavioral guidelines. These include demographic correlates, such as gender and age (Zettler et al., 2022), political orientation (Painter and Qiu, 2020; Gadarian et al., 2021), trust in science (Plohl and Musil, 2021), personality traits (e.g., Aschwanden et al., 2021; Blagov, 2021), thinking style (Thoma et al., 2021; Xu and Cheng, 2021), perceived fear of the virus (Harper et al., 2021), identification with humanity (Barragan et al., 2021), psychological entitlement (Li, 2021), self-efficacy (Chong et al., 2020; Jorgensen et al., 2021), and even cognitive factors, such as working memory capacity (Xie et al., 2020).

Previous attempts to explain individual differences in adherence to COVID-19 prevention guidelines have predominantly focused on intentional behaviors driven by motivational factors and attitudes. Consequently, the heterogeneity in compliance with the official prevention measures was explained in terms of a rational decision-making process (Xie et al., 2020; Thoma et al., 2021), which includes an explicit consideration of the implicated costs (e.g., reduced social and physical contact, the loss of freedom, and familiar routines) and benefits (e.g., avoiding contamination and mitigating the pandemic). For example, the greater tendency of women, as compared to men, to engage in preventive behaviors was ascribed to variance in the way the severity of COVID-19 was perceived by different genders, and to the greater obligation felt by women to protect others (Galasso et al., 2020; Paramita et al., 2021). The positive relationship between working-memory capacity and social distancing compliance was explained as contingent on one’s ability to compare multiple pieces of potentially conflicting information in working memory (Xie et al., 2020). Indeed, noncompliance could be due to an explicit decision to deviate from official prevention guidelines. As such, it might be related to a more general inclination towards deliberately violating rules. Nevertheless, if we focus narrowly on intentions *per se*, other potential predictors of precautionary behavior may be neglected. Noncompliance can take many forms, and could be shaped, not only by intentions, but also by involuntary errors due to flawed planning, failures of memory, or mal-attentional control (Ubani et al., 2020; Falco and Zaccagni, 2021; Thoma et al., 2021). Moreover, various categories of deliberate violations and various categories of errors could contribute to noncompliance.

An extensive literature documents a gap between intentions and actual behavior across a wide range of domains (Momsen and Stoerk, 2014; Zhang et al., 2019). Intentions to comply may fail to translate into corresponding actions, due to deficient planning, difficulties in carrying out planned behavior, forgetfulness, and poor estimation (Xie et al., 2020; Thoma et al., 2021). In particular, habit is an important factor relevant to the translation of intentions into behaviors (Sniehotta et al., 2005; Kothe et al., 2015). COVID-19 measures involve immediate and extensive deviation from automatic internalized daily routines and adoption of new behavioral habits (e.g., mask wearing, hands hygiene, and social distancing). Such behavioral changes tax attentional and working memory capacities (Lee et al., 2020) and are therefore difficult, even when the benefits are evident (Moya et al., 2020). Planning helps overcome forgetfulness and promotes recall of the intended behavior (Rogers et al., 2015). In the absence of a plan, individuals may be more prone to focus on low-effort/low-return tasks, falling short of their goals (Abel et al., 2019).

### Conscientiousness as a test case

Conscientiousness, which is part of the Five Factors Model (FFM) of personality traits (McCrae and Costa, 1987) is a broad construct describing the tendency to be self-controlled, responsible, industrious, orderly, well-organized, and rule-abiding (MacCann et al., 2009). Although various psychological constructs are linked in the literature to COVID-19 behavior, several considerations led us to focus on Conscientiousness as a test-case, for the utility of a multifaceted behavioral failure lens in illuminating pathways to adherence: (1) Conscientiousness stands out as one of the most frequently identified individual predictors of COVID-19 adherence behavior. Recent research has established that highly conscientious individuals are significantly more likely to abide to COVID-19 prevention guidelines (e.g., Asselmann et al., 2020; Clark et al., 2020; Nofal et al., 2020). It was also identified as an important predictor of other health-related behaviors (Tucker et al., 2006) and of safety behaviors (e.g., Lawton and Parker, 1998). (2) Despite considerable research demonstrating the association of Conscientiousness with adherence to COVID-19 guidelines and with other health behaviors, less is known about the mechanisms underneath these relationships (Tucker et al., 2006). (3) The broad nature of Conscientiousness implies that the higher adherence of conscientious individuals to the COVID-19 guidelines might be multiply determined. Hence, employing a multifaceted perspective of behavioral-failure to explore this relationship may enhance our understanding and guide constructive intervention strategies. For example, the greater compliance of conscientious individuals might be due to their greater tendencies toward responsibility and their higher likelihood of adhering to norms and rules (Carvalho et al., 2020; Clark et al., 2020; Aschwanden et al., 2021; Blagov, 2021; Zettler et al., 2022), but also due to their sense of organization and orderliness. The tendency of conscientious individuals to think ahead, make plans, and be firmly in control might help them in carrying out plans, persevere, and cope with obstacles (Keizer et al., 2019). These two accounts are by no means mutually exclusive.

### Behavioral failures perspective

Human behavioral failures result in fatalities, heavy economic loss, and emotional distress (Senders and Moray, 2020). Given the grave implications of COVID-19, noncompliance with prevention guidelines may be regarded as a specific type of behavioral-failure. Thus, a typology of behavioral-failure could prove useful in identifying distinct routes to noncompliance. The literature reviewed here suggests that a meaningful consideration of individual differences in adherence to COVID-19 prevention guidelines requires a multifaceted perspective, which incorporates various categories of both deliberate and unintended behaviors. Yet, relatively little research has directly examined the association between behavioral failures and COVID-19 precautionary behavior (Kleitman et al., 2021). The scarce research that has addressed this issue has employed a narrow perspective reflecting only a fraction of the possible varieties of behavioral failures. There is, for example, evidence that individual differences in cognitive failures [measured with the Cognitive Failures Questionnaire (CFQ); Broadbent et al., 1982], a trait-like construct that taps into the subjective experience of failures in attention, memory and motor function, is related to weaker adherence to COVID-19 precautionary measures (Thoma et al., 2021). Similarly, there is evidence that COVID-19 adherence behavior is related to judgement errors due to insufficient working memory capacity (Xie et al., 2020), and to ill-estimations (e.g., underestimating the risk of contracting COVID-19 from an asymptomatic coworker or friend; Advani et al., 2021).

A commonly used multifaceted framework for the research of behavioral-failure is the Generic Error Modeling System (GEMS) proposed by Reason (1990). Reason mainly differentiates between errors that are unintended and violations which are deliberate deviations from written prescribed rules and instructions. Reason further distinguishes between two kinds of errors: slips and lapses, which he defined as the unwitting deviation of action from intention (mostly due to failures of perception, attention, or memory) and mistakes, which he defined as the departure of planned actions from some satisfactory path toward the desired goal (mostly due to failures of judgement, estimation and decision; Reason et al., 1990, p. 1315–1316). This model has been proven useful in the research of traffic accidents and in a few additional domains, such as aviation (Wiegmann and Shappell, 2001) and rail transport (Free, 1994). A broad research program with the Driver Behavior Questionnaire (DBQ; Reason et al., 1990) showed that particular categories of behaviors serve as distinct pathways to accidents (e.g., Reason et al., 1990; Parker et al., 1995a,b). Yet, to the best of our knowledge, this typology has not yet been implemented in the research of health behaviors or COVID-19 behavior.

Recently, Diamant and Rusou (2021) developed and validated the Failures Proneness (FP) questionnaire, a brief, multifaceted, self-report scale to assess individual differences in the predisposition for behavioral failures in everyday settings. This scale comprises six distinct factors of behavioral-failure: *(1) Lapses (LP)* captures failures in attention, alertness, memory, and daydreaming, *(2) Disorganization-Errors (DE)* denotes one’s ability to organize, plan, and uphold daily routines, *(3) Temporal-Errors (TE)* depicts maladaptive time management failures in temporal estimation and judgment, *(4) Procedural-Violations (PV)* describes deviance from guidelines or regulations in order to promote other goals perceived as more valuable, *(5) Noncompliance-Violations (NV)* is associated with non-conforming attitudes and low internalization of norms, and *(6) Risks (RK)* is conceptually similar to sensation and risk seeking (Zuckerman, 2014), which is related to delinquency (Ljubin-Golub et al., 2017) and unsafe behavior (Zhang et al., 2019). This factor structure is congruent with Reason’s original theoretical distinction between lapses, mistakes, and violations (1990): *Lapses* resembles Reason’s notion of lapses and notion of cognitive failure of Broadbent et al. (1982); *Disorganization-Errors (DE)* and *Temporal-Errors* are congruent with Reason’s definition of mistakes, and *Procedural-Violations* and *Noncompliance-Violations* denote deliberate deviation from prescribed rules, and are therefore parallel to Reason’s definition of violations. FP expands this typology to everyday environments, and also distinguishes between different types of errors and different kinds of violations. Divergent relationships obtained between FFM personality traits and distinct categories of the FP (Diamant and Rusou, 2021) highlight its multifaceted nature and point to its potential ability to illuminate the pathways through which personality is associated with unsafe behavior. Hence, the FP could explain individual differences in adherence to COVID-19 measures and provide a powerful vehicle to assist in understanding the complex relationships between personality and COVID-19 behaviors.

### Current research

**Figure 1:**
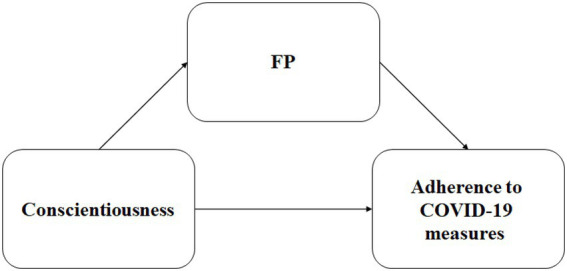
Conceptual mediation model.

This research employed a behavioral-failure lens to explore individual differences in adherence to COVID-19 prevention guidelines and the association between Conscientiousness and adherence (Clark et al., 2020; Aschwanden et al., 2021; Götz et al., 2021; Zettler et al., 2022). We hypothesized that the FP questionnaire would predict individual differences in nonadherence to COVID-19 measures (Hypothesis 1) and mediate the relationship between Conscientiousness and adherence (Hypothesis 2). Another aim of the research was to examine the relative ability of the FP questionnaire and the CFQ to predict noncompliance with precautionary measures. Since FP incorporates various categories of both intentional and unintentional behaviors, we assumed that it would predict noncompliance with precautionary measures over and above the CFQ, which includes merely unintentional slips and lapses (Hypothesis 3). Hypothesis 1 and Hypothesis 2 were tested in both studies. Hypothesis 3 was tested in Study 2 only (Figure 1).

## Study 1

Study 1 aimed to assess the relationship between Conscientiousness, COVID-19 prevention behavior, and a more general predisposition to behavioral failures. We hypothesized that individual-differences in the predisposition to behavioral failures will predict nonadherence to COVID-19 guidelines. Higher scores of FP will be accompanied by less adherence to the guidelines (Hypothesis 1). We also hypothesized that people higher in Conscientiousness would report a lower predisposition to behavioral failures and would be more likely to adhere to COVID-19 prevention measures (Hypothesis 2). The study was conducted during the peak of the third wave of the pandemic in Israel (through December 15–27, 2020 and February 7–March 9, 2021), when COVID-19 cases were steadily increasing in Israel, and as several countries announced the appearance of the new and more infectious Alpha and Beta COVID-19 strains. At that time, Israel’s vaccination program had just started, and had initially focused on the elderly (people above the age of 60) and individuals with a higher risk for severe illness. Since most of our participants were younger, the vast majority was not vaccinated at the time of the study, and the only reasonable way they had of avoiding infection was by adhering to preventative measures.

## Method

### Participants

A total sample of 752 individuals, aged 18–69, (*M* = 29.21, *SD* = 8.77, 75.1% women) participated in the study. Of these, 494 (65.7%) were undergraduate students from a university in central Israel, who participated in the study as part of their academic requirements, and 258 (34.3%) were volunteers recruited through social media, professional forums, and email by a snow-ball method. The inclusion criterion was age ≥ 18. Of the participants, 212 (28.2%) had an academic degree. A sensitivity analysis with G^*^Power (Faul et al., 2007) suggested that with *N* = 752, we have power = 1.00 to detect effect sizes ≥0.05 in Linear multiple regression; Fixed model single regression coefficient (*p* = 0.05).

### Measures

#### Failures proneness questionnaire

A brief, multifaceted self-report scale comprised of 16 items that assess the predisposition for behavioral failures in everyday settings. The scale incorporates six factors: Lapses (LP) which captures failures in attention, alertness, memory, and daydreaming (e.g., “I have difficulty in following what is said in a meeting with numerous participants”), Disorganization-Errors (DE) which denotes general disorganization and failures in estimation and judgment (e.g., “I am punctilious and precise in what I do”), Temporal-Errors (TE) which depicts maladaptive time management (e.g., “I tend to be late or to arrive at the last moment”), Procedural-Violations (PV) which describes deviance from guidelines or regulations in order to promote other goals perceived as more valuable (e.g., “I tend to “round corners” in order to further my work and to finish on time”), Noncompliance-Violations (NV) which is associated with non-conforming attitudes and low internalization of norms (e.g., “I tend to attach utmost importance to adherence to rules and directives”), and Risks (RK), which denotes sensation and risk seeking, (e.g., “I like doing things that lead to suspense and excitement”). Participants were asked to indicate their experience with each item on a Likert scale, ranging from 1 (never), to 7 (very often).

#### Adherence to COVID-19 behavioral measures

To measure participants’ adherence to COVID-19 measures, we used the guidelines issued by the Israeli Ministry of Health (e.g., wearing a face mask and social distancing) and questionnaires presented by previous studies (Bogg and Milad, 2020; Nudelman et al., 2021). The scale consisted of 12 items. Respondents were asked to rate their adherence to each of these guidelines during the previous week, on a five-point Likert scale ranging from 1 (never) to 5 (always). A total adherence score was computed by averaging the items.

#### Conscientiousness

We used a nine-item scale (*α* = 0.73) taken from the Hebrew short version (Etzion and Laski, 1998) of the 44-item Big Five Inventory (BFI; John et al., 2008) of personality traits (McCrae and Costa, 1987). The items were rated by participants on a five-point Likert scale ranging from 1 (strongly disagree) to 5 (strongly agree).

### Procedure

The study was conducted online using Qualtrics. Informed consent was obtained before data collection. Participants first completed the *FP*, then the *BFI*, and finally the Adherence to the COVID-19 Preventive Measures questionnaire. Age and gender were also indicated. All questionnaires were administered in a single session.

### Data analysis

Means, SDs, category frequencies, and percentages, and Pearson correlations were calculated for the background and research variables. The ability of the FP subscales to predict compliance to COVID-19 measures was evaluated using hierarchical regression models, with demographic predictors (age and gender) being included in block 1 and the six subscales of FP being included at block 2. Finally, the effect sizes of the mediation models were assessed using Hayes’ PROCESS 3.5 macro in SPSS V.26 (model 4) with Conscientiousness as independent variable, gender, and age as covariates, the FP as mediators, and adherence to COVID-19 as the dependent variable. To explore whether one or more subscales mediated this relationship, we also ran a parallel mediation analysis model using the SPSS Process Macro Version 3.5 (Hayes, 2017) based on 5,000 bootstrap samples. Similar to the previous model, Conscientiousness was the independent variable, gender and age were covariates, adherence to COVID-19 measures as the dependent variable, however, we replaced the FP index with all its six subscales (LP, NV, PV, DE, TE, and Risks) as mediators.

To test for the underlying regression assumptions (multicollinearity, normality of residuals, linearity, and homoscedasticity), complementary regression models were run in SPSS V.26 (collinearity diagnostics, the Durbin-Watson test and visual inspection of relevant plots).

Since our analyses relied on self-reported data, it may be sensitive to common method variance (CMV; Podsakoff et al., 2003), which refers to *spurious correlations between study variables* due to a variety of response biases that can occur when similar methods (e.g., self-report) are used to collect data on different outcome measures. To assess common method bias, we performed the Harman’s single factor test by entering all items of the corresponding study variables (FP, COVID 19 guideline adherence, and Conscientiousness) into a principal component analysis (PCA).

### Results and discussion

Table 1 presents the first-order correlations between all study variables: gender, age, Conscientiousness, Failure Proneness (with its six sub-scales), and adherence to the COVID-19 behavioral measures, as well as means, SDs, and Cronbach’s *α* reliabilities for all primary study variables.

**Table 1:** Means, SDs, Cronbach’s α reliabilities, and Pearson’s *r* correlation for the variables measured in Study 1.

	*M*	*SD*	α	Gender^a^	Age	C	LP	DE	TE	PV	NV	RK	FP
Age	29.21	8.8		−0.01									
C	3.90	0.57	(0.73)	−0.14^**^	0.17^**^								
LP	3.91	1.16	(0.65)	−0.10^**^	−0.18^**^	−0.43^**^							
DE	3.00	1.29	(0.48)	0.13^**^	−0.06	−0.56^**^	0.18^**^						
TE	3.19	1.65	(0.79)	0.00	−0.13^**^	−0.32^**^	0.34^**^	0.22^**^					
PV	3.33	1.30	(0.69)	0.12^**^	−0.04	−0.44^**^	0.31^**^	0.30^**^	0.25^**^				
NV	2.78	1.10	(0.62)	0.20^**^	−0.09^*^	−0.37^**^	0.09^*^	0.38^**^	0.17^**^	0.32^**^			
RK	4.16	1.41	(0.67)	0.16^**^	−0.12^**^	−0.08^*^	0.13^**^	0.03	0.18^**^	0.23^**^	0.11^**^		
FP	3.42	0.77	(0.77)	0.12^**^	−0.18^**^	−0.62^**^	0.66^**^	0.54^**^	0.61^**^	0.70^**^	0.55^**^	0.44^**^	
CVD	3.48	0.87	(0.90)	−0.22^**^	0.14^**^	0.24^**^	−0.10^**^	−0.22^**^	−0.15^**^	−0.23^**^	−0.40^**^	−0.18^**^	−0.35^**^

* *p* < 0.05;

** *p* < 0.01.

α-Cronbach reliabilities in parentheses; C, Conscientiousness; LP, Lapses; DE, Disorganization-Errors; TE, Temporal-Errors; PV, Procedural-Violations; NV, Noncompliance-Violations; RK, Risks; FP, Failure Proneness; and CVD, COVID-19 guideline adherence.

a “female” is coded as “0” and “male” is coded as “1.” Hence, a positive correlation indicates a higher score for men, and a negative correlation indicates a higher score for women.

While the self-reported behaviors exhibited a moderate degree of compliance to the COVID-19 guidelines, the presence of individual differences suggests that an investigation of the factors underlying this variability is warranted.

#### Demographic correlates

Several studies have reported that women exhibit higher adherence to COVID-19 precautionary measures (Clark et al., 2020) and that compliance increases with age. Similarly, different types of behavioral failures have different demographic correlates, with men reporting more violations than women, while women describe more lapses. Additionally, violations (but not lapses) tended to decrease with age (de Winter et al., 2015). Hence, we included gender and age in the analysis. As Table 1 shows, women were more likely than men to adhere to COVID-19 guidelines (negative *r* values) and adherence increased with age. As to failures, men scored significantly higher on Procedural-Violations, Noncompliance-Violations, Risks and Disorganization-Errors, and had a higher FP total score (positive *r* values), while women scored significantly higher on Lapses (negative *r* values). Gender differences for Temporal-Errors were not significant. Lapses, Temporal-Errors, Noncompliance-Violations, Risks, and the total FP score decreased with age.

#### Relationship between behavioral failures and COVID-19 adherence behavior

A major aim of the research was to assess the relationship between COVID-19 prevention behavior and a more general predisposition to behavioral failures (Hypothesis 1). To this end, we calculated the bivariate correlation between self-reported adherence to COVID-19 measures and FP. As can be seen in Table 1, all FP subscales were negatively correlated with COVID-19 adherence behavior: individuals who were more prone to failures were less likely to adhere to COVID-19 guidelines. The association with Noncompliance-Violation (NV) was the strongest, followed by medium sized associations with Disorganization-Errors (DE) and with Procedural-Violations (PV), and the rest of the factors being weakly associated with COVID-19 adherence behavior.

Next, to assess the unique contribution of each first-order factor of FP to (non)adherence behavior, FP’s subscales were regressed on COVID-19 adherence behavior together with age and gender in a hierarchical regression model. Preliminary analyses were performed to ensure there was no violation of normality, linearity, homoscedasticity (all three with visual inspection of relevant plots) multicollinearity (*VIF <* 1.33), and correlations of residuals (*Durbin-Watson* = 1.96). As can be seen in Table 2, only Noncompliance-Violations (*β* = −0.31, *p* < 0.001) and Risks (*β* = −0.08, *p* = 0.02) significantly predicted COVID-19.

**Table 2:** Results of hierarchical multiple regressions for COVID-19 adherence behavior (Study 1).

Variables	*B*	SE B	*β*	*t*	Sig.	$R^{2}$	$\Delta R^{2}$
Step 1	3.197	0.108		29.507	0.000^***^	0.069	
Gender	−0.449	0.071	−0.223	−6.304	0.000^***^		
Age	0.014	0.003	0.137	3.868	0.000^***^		
Step 2	4.553	0.186		24.433	0.000^***^	0.207	0.146
Gender	−0.270	0.069	−0.134	−3.923	0.000^***^		
Age	0.009	0.003	0.086	2.567	0.010^*^		
Lapses	−0.013	0.027	−0.017	−0.464	0.643		
Disorganization-Errors	−0.030	0.025	−0.045	−1.218	0.224		
Temporal-Errors	−0.021	0.019	−0.039	−1.096	0.273		
Procedural-Violations	−0.046	0.025	−0.069	−1.846	0.065		
Noncompliance-Violations	−0.244	0.029	−0.310	−8.407	0.000^***^		
Risks	−0.051	0.021	−0.083	−2.419	0.016^*^		

SE, Std. Error.

* *p* < 0.05 and

*** *p* < 0.001.

#### Adherence to COVID-19 guidelines and Conscientiousness

**Figure 2:**
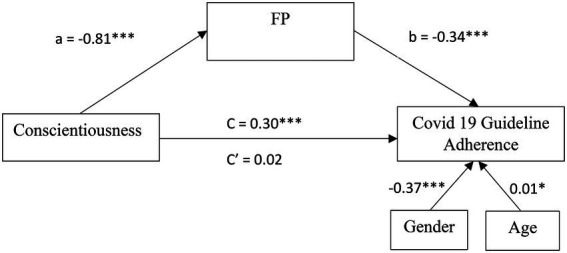
Mediation model of FP for the relationship between Conscientiousness and COVID 19 Guideline Adherence in Study 1. All values are unstandardized coefficients ^*^p < 0.05 and ^***^p < 0.001.

A weak positive correlation was found between Conscientiousness and compliance to COVID-19 measure [*r*_(747)_ = 0.24, *p* < 0.001]. Participants who scored higher in Conscientiousness were more inclined to follow COVID-19 guidelines. To test whether FP mediate the relationship between Conscientiousness and adherence (Hypothesis 2), we ran a mediation model with Conscientiousness as independent variable, gender and age as covariates, FP as mediators, and adherence to COVID-19 measures as the dependent variable, using the SPSS Process Macro Version 3.5 (Hayes, 2017) based on 5,000 bootstrap samples. Preliminary analyses were performed to ensure there was no violation of normality, linearity, homoscedasticity (all three with visual inspection of relevant plots) multicollinearity (*VIF* = 1.63), and autocorrelation (*Durbin-Watson* = 1.97). Figure 2 presents a summary of the analysis.

The results show that Conscientiousness significantly predicted COVID-19 adherence behavior [*c* = 0.30, *SE* = 0.05, *β* = 0.20, *F*(1,745) = 31.06, *p* < 0.001]. This relationship was fully mediated by FP (a^*^b = 0.28, *SE* = 0.04, CI_95_[0.20, 0.37], *β* = 0.18, *p* < 0.001). Conscientiousness significantly predicted FP [*a* = −0.81, *SE* = 0.04, *β* = −0.61, *F*(1,745) = 429.82, *p* < 0.001] and FP significantly predicted adherence behavior [*b* = −0.34, *SE* = 0.05, *β* = −0.35, *F*(1,745) = 48.78, *p* < 0.001]. FP accounted for approximately 90% of the total effect of Conscientiousness on adherence behavior [*P_M_* = (0.18)/(0.20)]. After controlling for the mediator, Conscientiousness was no longer a significant predictor of adherence behavior (*c*’ = 0.02, *SE* = 0.07, *β* = 0.02, *t* = 0.35, ns).

We then explored whether one or more subscales mediated the relationship between Conscientiousness and COVID-19 adherence behavior. For this purpose, we ran a parallel mediation analysis model using the SPSS Process Macro Version 3.5 (Hayes, 2017) based on 5,000 bootstrap samples. Similar to the previous model, Conscientiousness was the independent variable, gender and age were covariates, adherence to COVID-19 measures as the dependent variable, however, we replaced the FP index with all its six subscales (LP, NV, PV, DE, TE, and Risks) as mediators. Preliminary analyses were performed to ensure there was no violation of normality, linearity, homoscedasticity (all three with visual inspection of the relevant plots) multicollinearity (all *VIF*s < 2) and autocorrelation (*Durbin-Watson* = 1.96). Table 3 presents a summary of the standardized indirect effects and their CIs.

**Table 3:** Completely standardized indirect effects of Conscientiousness on COVID 19 Guideline Adherence.

Mediator Variables (M1–M6)	*β*	*SE*1	95% CI for β1	
			LL	UL
(TOTAL)	0.18	0.04	0.10	0.25
M1: Lapses	0.01	0.02	−0.03	0.04
M2: DE	0.02	0.03	−0.03	0.07
M3: TE	0.01	0.01	−0.01	0.04
M4: PV	0.03	0.02	−0.01	0.06
M5: NV	0.11	0.02	0.07	0.15
M6: Risks	0.00	0.00	−0.00	0.01

C, Conscientiousness; DE, Disorganization Errors; TE, Temporal Errors; PV, Procedural Violations; NV, Noncompliance-Violations; CI, confidence interval; LL, lower limit; UL, upper limit; 1 SE and CI based on 5,000 bootstrap resamples of the sample size (747) with replacement.

**Figure 3:**
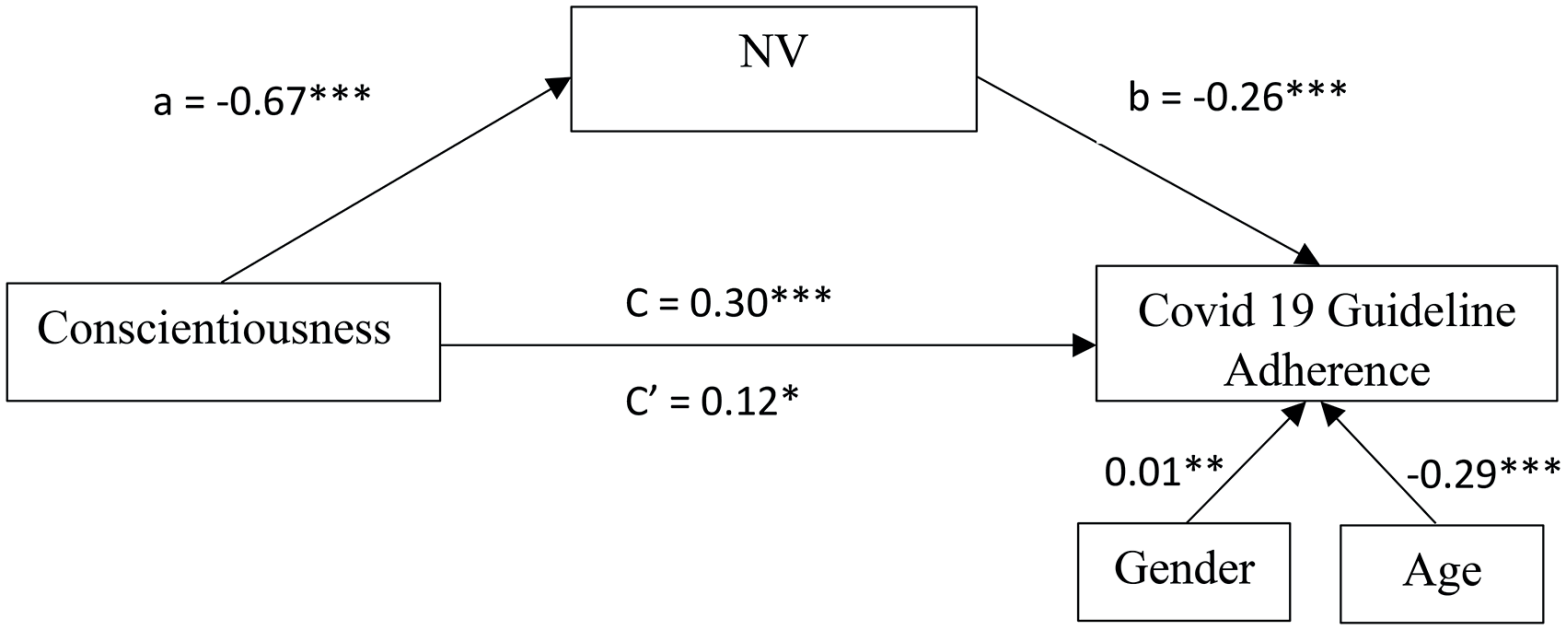
Mediation model of NV for the relationship between Conscientiousness and COVID 19 Guideline Adherence in Study 1. All values are unstandardized coefficients ^*^p < 0.05, ^**^p < 0.01, and ^***^p < 0.001.

Results from a parallel mediation analysis indicated that Conscientiousness is indirectly related to COVID 19 adherence behavior through its relationship with the NV subscale of FP. A CI based on 5,000 bootstrap samples indicated that the indirect effect through NV (a5b5 = 0.11), holding all other mediators, constant, was entirely above zero [0.07, 0.15]. All other subscales’ CIs were not different than zero and therefore had no significant contributions to the indirect effect. Following this exploratory analysis, we ran another mediation model, this time only with the NV as a mediator. Figure 3 presents a model summary of the direct, indirect and total effects of this model.

First, as can be seen in Figure 3, higher levels of Conscientiousness were related to lower Noncompliance-Violation (*a =* −0.67; *SE* = 0.07; CI_95_[−0.80, −0.54]; *β* = −0.35; *p* < 0.001), and lower Noncompliance-Violation was subsequently related to more COVID-19 adherence behavior (*b =* −0.26; *SE* = 0.03; CI_95_[−0.32, −0.21]; *β* = −0.33; *p* < 0.001). Noncompliance-Violation significantly mediated the indirect effect between Conscientiousness and *COVID-19* adherence behavior with bootstrapped CI which above zero (*ab* = 0.18; *SE* = 0.03; CI_95_[0.12, 0.24]; *β* = 0.12) was entirely above zero. Finally, the direct effect of Conscientiousness on COVID-19 adherence behavior was weak but significant (*c* = 0.12; *SE* = 0.03; CI_95_[0.02, 0.23]; *β* = 0.08; *p* = 0.02). Noncompliance-Violations, accounted for approximately 60% of the total effect on adherence behavior [*P_M_* = (0.12)/(0.20)]. These results indicate a partial mediation model.

#### Common method variance

The generated PCA output revealed nine distinct factors accounting for 58.3% of the total variance. The first unrotated factor captured only 20.6% of the variance in the data. Thus, no single factor emerged, and the first factor did not capture most of the variance. Therefore, these results suggested that no single factor explained the majority of variance in the data.

In sum, in accordance with our hypotheses, FP predicted individual differences in adherence to COVID-19 measures, and mediated the relationship between Conscientiousness and adherence behavior. Additionally, the parallel mediation model showed that among the six subscales of FP, only NV significantly mediated the relationship between Conscientiousness and COVID-19 adherence behavior. However, while the total FP score fully mediated this relationship, NV provided only a partial mediation.

## Study 2

Study 2 aimed at examining the relative ability of the FP and the CFQ to predict noncompliance with precautionary measures, and to provide a replication of Study 1 in a different context (a different phase of the pandemic). The study was conducted between May and July 2021. At this time, more than 50% of the population in Israel had been vaccinated and many had recovered from COVID-19. The number of new cases dropped, and the government gradually lifted or relaxed many of its COVID-19 guidelines (e.g., the requirement for mask wearing outdoors). We expected a reduction in adherence to COVID-19 measures (as compared to study 1) due to vaccinations and the decrease in the number of cases and deaths, as well as pandemic fatigue (Petherick et al., 2021). Yet, we hypothesized that the FP questionnaire would predict noncompliance with the precautionary measures (Hypothesis 1) over and above the CFQ (Hypothesis 3).

## Method

### Participants

A total sample of 482 undergraduate psychology students from a university in central Israel participated in the study as part of their academic requirements (ages between 18 and 61, *M* = 31.05, *SD* = 9.14, 82.4% women). A sensitivity analysis with G^*^Power (Faul et al., 2007) suggested that with *N* = 482, we have power = 1.00 to detect effect sizes ≥0.05 in Linear multiple regression; Fixed model single regression coefficient (*p* = 0.05).

### Measures

#### Failures proneness questionnaire

Same as in Study 1.

#### Cognitive failures questionnaire

The 25-item (Broadbent et al., 1982) questionnaire assesses one’s proneness for committing failures in perception, memory and motor function in everyday common life (e.g., “Do you read something and find you have not been thinking about it and must read it again?”). Participants were asked to rate how often they have experienced these situations in the past 6 months, on a scale from 1 (*never*) to 5 (*very often*). The total score was the mean of the 25 items.

#### Adherence to COVID-19 behavioral measures

Same as in Study 1 was used. However, since at the time of the study, many COVID-19 measures had been lifted or eased by the government, respondents were asked to rate their adherence to each of these guidelines during the previous couple of months (rather than weeks).

#### Conscientiousness

Same as in Study 1.

### Procedure

The study was conducted online using Qualtrics. Informed consent was obtained before data collection. Participants first completed the FP, then the CFQ, then the BFI, and finally the adherence to COVID-19 Behavioral Measures. Age and gender were also indicated. All questionnaires were administered in a single session.

### Data analysis

Same as in study 1.

### Results and discussion

Table 4 presents the first-order correlations between all study variables: gender, age, Conscientiousness, Failure Proneness (with its six sub-scales), CFQ, and adherence to the COVID-19 behavioral measures, as well as means, SDs, and Cronbach’s α reliabilities for all primary study variables.

**Table 4:** Means, SDs, Cronbach’s α reliabilities, and Pearson’s *r* correlation for the variables measured in Study 2.

	*M*	*SD*	α	Gender^a^	Age	C	LP	DE	TE	PV	NV	RK	FP	CVD
Age	31.05	9.14		−0.10^*^										
C	3.86	0.60	(0.74)	−0.03	0.18^**^									
LP	3.85	1.24	(0.71)	−0.04	−0.16^**^	−0.50^**^								
DE	2.99	1.34	(0.51)	0.03	−0.06	−0.43^**^	0.17^**^							
TE	3.15	1.65	(0.76)	0.07	−0.08	−0.40^**^	0.45^**^	0.18^**^						
PV	3.50	1.32	(0.68)	0.09^*^	−0.12^*^	−0.39^**^	0.36^**^	0.19^**^	0.27^**^					
NV	2.84	1.25	(0.64)	0.12^*^	−0.08	−0.31^**^	0.11^*^	0.44^**^	0.16^**^	0.17^**^				
RK	3.93	1.47	(0.65)	0.15^**^	−0.13^**^	−0.15^**^	0.23^**^	−0.03	0.32^**^	0.32^**^	0.13^**^			
FP	3.41	0.82	(0.78)	0.10^*^	−0.18^**^	−0.61^**^	0.72^**^	0.49^**^	0.65^**^	0.66^**^	0.54^**^	0.51^**^		
CVD	3.55	0.94	(0.93)	−0.23^**^	0.13^**^	0.12^**^	−0.11^*^	−0.16^**^	−0.05	−0.12^*^	−0.35^**^	−0.13^**^	−0.25^**^	
CFQ	3.36	0.66	(0.92)	−0.09	−16^**^	−0.56^**^	0.71^**^	0.20^**^	0.42^**^	0.37^**^	0.15^**^	0.22^**^	0.61^**^	0.08

* *p* < 0.05;

** *p* < 0.01.

α-Cronbach reliabilities in parentheses; C, Conscientiousness; LP, Lapses; DE, Disorganization-Errors; TE, Temporal-Errors; PV, Procedural-Violations; NV, Noncompliance-Violations; RK, Risks; FP, Failure Proneness; and CVD, COVID-19 guideline adherence.

a “female” is coded as “0” and “male” is coded as “1.” Hence, a positive correlation indicates a higher score for men, and a negative correlation indicates a higher score for women.

#### Demographic correlates

As Table 4 shows, women scored significantly higher than men on adherence to COVID-19 guidelines (negative *r* values). Men scored significantly higher on Procedural-Violations, Noncompliance-Violations and Risks, and had a higher FP total score (positive *r* values), but no gender differences were found for Lapses, Disorganization-Errors, and Temporal-Errors, or Cognitive-Failure. Voluntary compliance behaviors increased with age, and Cognitive-Failure, Lapses, Procedural-Violations, Risks, and total FP score decreased with age.

#### The relationship between behavioral failures and COVID-19 adherence behavior

As Table 4 shows, in accordance with the first hypothesis, participants who were more prone to failures (had higher FP scores) were less inclined to follow COVID-19 guidelines. COVID-19 adherence behavior was moderately correlated with Noncompliance-Violations, and weakly correlated with Lapses, Disorganization-Errors, and Procedural-Violations and Risks. However, CFQ scores were not associated with COVID-19 adherence behavior. Thus, the third hypothesis was also supported.

Next, FP’s subscales were regressed on COVID-19 adherence behavior together with age and gender in a hierarchical regression model. Preliminary analyses were performed to ensure there was no violation of normality, linearity, homoscedasticity (all three with visual inspection of relevant plots), multicollinearity (*VIF* < 1.41), and autocorrelations (*Durbin-Watson* = 1.80). Results showed only Noncompliance-Violations (*β* = −0.31, *p* < 0.001) significantly predicted COVID-19 behavior (Table 5).

**Table 5:** Results of hierarchical multiple regressions for COVID-19 adherence behavior (Study 2).

Variables	*B*	SE B	*β*	*t*	Sig.	*R^2^*	$\Delta R^{2}$
Step 1	3.322	0.152		21.916	0.000^***^	0.065	
Gender	−0.549	0.110	−0.222	−4.997	0.000^***^		
Age	0.011	0.005	0.103	2.317	0.021^*^		
Step 2	4.396	0.245		17.937	0.000^***^	0.176	0.111
Gender	−0.473	0.106	−0.192	−4.457	0.000^***^		
Age	0.007	0.004	0.066	1.532	0.126		
Lapses	−0.072	0.038	−0.095	−1.911	0.057		
Disorganization-Errors	−0.009	0.034	−0.013	−0.270	0.787		
Temporal-Errors	0.045	0.028	0.078	−1.597	0.111		
Procedural-Violations	−0.003	0.034	−0.004	−0.091	0.927		
Noncompliance-Violations	−0.233	0.036	−0.309	−6.466	0.000^***^		
Risks	−0.033	0.030	−0.051	−1.096	0.274		

SE, Std. Error.

* *p* < 0.05 and

*** *p* < 0.001.

#### Adherence to COVID-19 prevention guidelines and Conscientiousness

**Figure 4:**
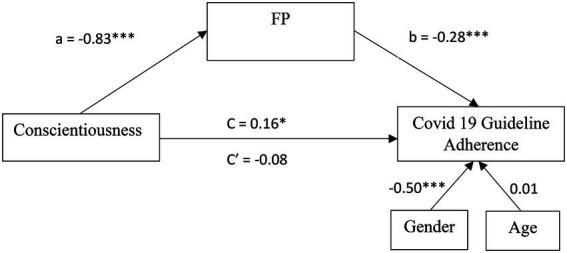
Mediation model of FP for the relationship between Conscientiousness and COVID 19 Guideline Adherence in Study 2. All values are unstandardized coefficients ^*^p < 0.05 and ^***^p < 0.001.

**Figure 5:**
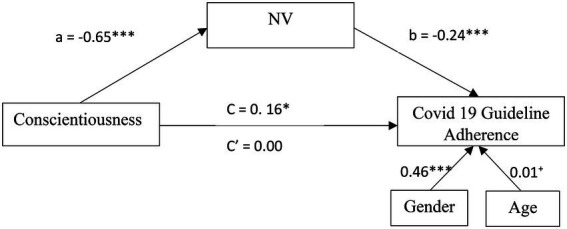
Mediation model of NV for the relationship between Conscientiousness and COVID 19 Guideline Adherence in Study 2. All values are unstandardized coefficients ^+^*p* = 0.055, ^*^p < 0.05, and ^***^p < 0.001.

A weak positive correlation was found between Conscientiousness and adherence to COVID-19 measures [*r*_(480)_ = 0.12, *p* = 0.01]. Participants who scored higher on Conscientiousness were more inclined to follow COVID-19 guidelines. To test the second hypothesis, mediation analyses replicating study 1’s model, were performed with the same methodology (mediation for FP, parallel mediation for all subscales of FP, and mediation for the only confirmed mediator—NV). Preliminary analyses of all models found no violations of any of the assumptions reported in study 1 (all *VIF* < 1.78; *Durbin-Watson* = 1.80). Figure 2 presents a summary of the analysis. Findings successfully replicated both mediation paths of FP in the first analysis (Figure 4) followed by the exploratory parallel mediation analysis of FP’s six subscales. Again, only NV had a mediation path for the relationship between Conscientiousness and COVID19 adherence behavior (Figure 5), although all effects were slightly weaker in study 2, full mediation was detected for both the FP mediation model (a^*^b = 0.24, *SE* = 0.06, CI95% [0.13, 0.35], *β* = 0.15) and the NV mediation model (a^*^b = 0.16, *SE* = 0.03, CI95% [0.10, 0.23], *β* = 0.10).

#### Common method variance

The generated PCA output revealed 14 distinct factors accounting for 61.8% of the total variance. The first unrotated factor captured only 21.5% of the variance in the data. Thus, no single factor emerged, and the first factor did not capture most of the variance. Therefore, these results suggested that no single factor explained the majority of variance in the data.

To summarize, Study 2 corroborated the patterns of Study 1 and demonstrated that the FP questionnaire predicts noncompliance with precautionary measures, over and above the CFQ.

## General discussion

As the COVID-19 outbreak has demonstrated, human behavior plays a crucial role in the propagation of pandemics. The present research investigated individual differences in COVID-19 precautionary behavior through a multifaceted lens of behavioral-failure—a framework that, to the best of our knowledge, has not been used to explore pandemic behavior or health behavior in general. Together, the two studies reported here provide strong support for the hypothesis that individual variations in the predisposition to behavioral failures predict (non)compliance with COVID-19 measures (Hypothesis 1). People who reported greater incidences of behavioral failures also reported less adherence to precautionary measures. While the Failure Proneness (FP) questionnaire, which incorporates several distinct factors of behavioral-failure, has consistently predicted compliance with COVID-19 guidelines while Cognitive Failure Questionnaire (CFQ), which denotes merely slips and lapses in cognitive functioning, did not (in line with Hypothesis 3). FP also mediated the previously established relationships between Conscientiousness and COVID-19 adherence behavior (in line with Hypothesis 2).

### FP and adherence with COVID-19 guidelines

A closer scrutinization of the relationship between adherence to the prevention guidelines and each of the first-order factors of FP highlights several interesting patterns: The results from the first-order correlation show that all FP subscales were negatively and significantly correlated with COVID-19 adherence behavior. The association with Noncompliance-Violation (NV) was the strongest, followed by a moderate association with Disorganization-Errors (DE) and with Procedural-Violations (PV), with the rest of the factors being weakly associated with COVID-19 adherence behavior. This pattern was consistent across the two studies (except the effect of Temporal-Errors which was not significant in Study 2). Nevertheless, in the regression analysis, COVID-19 behavior was predicted only through the intentional factors (and mainly by Noncompliance-Violations). The individual contribution of nonintentional behavioral-failures (as measured by the Lapses, Disorganization-Errors, and Temporal-Errors subscales) observed in the first-order correlations were not significant anymore. Hence, our data lend support to the notion that noncompliance with official COVID-19 prevention guidelines is driven mainly by intentional factors related to violation of norms and rules.

The apparent relationship between nonadherence to COVID-19 prevention guidelines and Noncompliance-Violations draws attention to the close linkage between violating COVID-19 guidelines and the broader phenomenon of rule violation (i.e., deliberate deviations from the prescribed rules), and brings to light the contribution of nonconformity and disrespect for rules. This pattern substantiates previous views of COVID-19 precautionary behavior as intentional and as involving deliberate considerations (Xie et al., 2020; Thoma et al., 2021). Rule violation is often seen as a consequence of a conflict between the micro level of an individual who is attempting to optimize behavior and the macro level of a society (or an organization), which attempts to control and constrain the behavior of its members (Lawton, 1998). At the micro level, a particular precautionary behavior would be perceived as suboptimal for an individual when its costs are perceived as outweighing the benefits. Yet, at the macro level, such behavior performed by all members in a society (or organization) is beneficial, as it serves to minimize losses to production or accidents (Battmann and Klumb, 1993; Lawton, 1998). Lawton (1998, p. 89) proposes driving in excess of the speed limit as an example:

“The penalties associated with speeding (e.g., fines and accidents) are unpredictable and rare, but the benefits (getting to your destination quicker, thrill etc.), are immediate and frequent. While for society the economic and social costs of road traffic accidents are huge, from an individual’s perspective the costs appear unlikely and distant when compared to the tangible and immediate benefits.”

In the same vein, the personal consequences of infection with COVID-19 are generally perceived to be minor, and hence the risk of ignoring precautions appears low to the individual. At the community level, however, successful mitigation of the disease critically relies on people’s voluntary compliance with recommended guidelines. The more the individual risk is combined, the greater the community risk becomes (Xie et al., 2020; Zivin and Sanders, 2020).

Since the Noncompliance-Violations subscale is related to various deviant behaviors, such as driving under the influence of alcohol and drug use (Diamant and Rusou, 2021), our findings strengthen and extend previous evidence linking nonadherence to the COVID-19 guidelines to individual characteristics, such as antisocial potential, low acceptance of moral rules, legal cynicism, low shame or guilt, engagement in delinquent behaviors, association with delinquent peers, and reporting high levels of antisociality (O’Connell et al., 2020; Zajenkowski et al., 2020; Miguel et al., 2021; Nivette et al., 2021; Zettler et al., 2022). The association between nonadherence to COVID-19 guidelines and Noncompliance-Violations corroborate assertion of O’Connell et al. (2020), that a behavior that increases risk of disease transmission is a specific form of antisociality.

The finding that intentional violations serve as precursors of nonadherence, while nonintentional errors (like Disorganization-Errors) do not, corroborate previous patterns manifested in the literature on traffic accidents, and substantiate the significance of violations in accident causation, which has been demonstrated repeatedly in traffic accident analysis (Lawton, 1998). Throughout this literature, different subscales of the Driver Behavior Questionnaire (DBQ; Reason, 1990) have been shown to be related to different actual dangerous road behaviors (observed in driving-simulators and real highways). In particular, certain dangerous road behaviors were significantly correlated with the violation subscale of the DBQ but not with the lapses or errors subscales. For example, the DBQ violation subscale was associated with objectively-measured speed (driving in excess of the speed limit), while the error and lapse sub-scales were not. Other driving behaviors that were found to be associated exclusively with violations are: frequent change of lanes, spending time in the left lane, sudden unidirectional accelerations, and accepting shorter inter-vehicle gap times (of approaching traffic) on left turns (Schwebel et al., 2006; Wallén Warner, 2006; De Winter et al., 2009; Stephens and Groeger, 2009; Zhao et al., 2012; Helman and Reed, 2015). Hence, the finding that only Noncompliance-Violations serve as a precursor of nonadherence to the COVID-19 guidelines, while other subscales have no explanatory power and corroborate previous patterns manifested in the literature on traffic accidents, in which a single subscale in a multifaceted behavioral-failure scale predicts a specific unsafe behavior.

Although we could expect that individuals who are predisposed to disorganization errors will face a greater difficulty in meeting the challenge of adhering to the COVID-19 guidelines, this association was evident only in the first-order correlations and was not significant in the partial regression analysis. This pattern is in accordance with numerous studies in the domain of traffic accidents which have indicated that self-reported violations were found to be a statistically significant, positive predictor of traffic accident involvement, while the self-reported tendency to make errors was not (Parker et al., 1995a,b). Nevertheless, disorganization errors denote poor planning and organization practices, and are related in the literature to the ability to transform intentions into actions. Hence, it is reasonable to assume that a predisposition to such errors would impede the deviation from automatic internalized daily routines and the adoption of new behavioral habits dictated by the COVID-19 measures (e.g., mask wearing, hands hygiene, and social distancing). Although our data does not enable to portray an effect of Disorganization-Errors, it is worth disentangling its unique contribution to non-adherence. The penalties of planning and organization difficulties were scarcely investigated in the context of the COVID-19 pandemic. We leave this important task for future research.

The correlation between non adherence to the guidelines and a more general predisposition to rule violations has obvious implications for devising interventions aimed at improving precautionary behavior. If unsafe COVID-19 behavior was the outcome of errors, then interventions such as reminders and planning aids may be successful in reducing such errors and enhancing compliance. Such interventions would have no influence on the commission of violations, which must be addressed by campaigns focusing on changing attitudes, increasing awareness to the potential consequences of behavior, and ensuring that the guidelines represent optimal behavior (in terms of costs and benefits at the micro and macro levels), as well as avoidance of unrealistic demands and increased enforcement (Lawton et al., 1997). In particular, noncompliance related to normative violations might be less malleable (Na and Paternoster, 2012). Yet, some might be tempered by rigorous monitoring and nudge (Nivette et al., 2021).

### Conscientiousness and adherence with COVID-19 guidelines

The current research heeds calls in the literature to expand our understanding of what underlies the linkage between personality (and Conscientiousness in particular) and behaviors that affect public health. Indeed, despite considerable research demonstrating consistent association between Conscientiousness, adherence to COVID-19 guidelines and other health-related behaviors, understanding of what underlies these relations is limited (Tucker et al., 2006). The two studies reported here demonstrate that a multifaceted behavioral-failure lens could illuminate the processes underlying the relationship between Conscientiousness and adherence to COVID-19 guidelines. And hence, this research may serve as one step toward such an understanding. Nevertheless, irresponsible behavior related to COVID-19 represents only one way in which people risk their own health and the health of others. Employing a multifaceted perspective of behavioral-failure to explore additional health-related behaviors may enhance our understanding of how personality traits are related to engagement in health-related behaviors and guide constructive intervention strategies.

### Comparative data of adherence to the COVID-19 guidelines across studies

As the COVID-19 pandemic lingered, the possibility of “pandemic fatigue” has raised worldwide concerns. Several studies have reported empirically meaningful and geographically widespread changes in adherence to COVID-19 guidelines over time (Petherick et al., 2021). Since there was a time gap between the two studies reported here, we have examined whether such temporal changes emerged in our data (Table 6). Although we expected a decline in adherence (in Study 2 as compared to Study 1), our data showed no such decrease in any of the COVID-19 measures. In both studies, self-reported behaviors exhibited a moderate degree of compliance. This pattern could reflect a decline decelerated over-time (Petherick et al., 2021) or changes in the reference points of our participants. Study 1 was conducted at the peak of the third wave of the pandemic, when COVID-19 cases were steadily increasing in Israel, the vast majority of the population was not vaccinated and the focus was strictly on reducing contact, infections, and death rates. In contrast, Study 2 was conducted 5 months later, between waves, when many people had been vaccinated, the number of COVID-19 cases was low, and the Israeli government gradually lifted or eased many of its guidelines for mask-wearing and social distancing. It may be the case that even though our participants practiced fewer precautionary behaviors, they still felt that they were complying with the guidelines.

**Table 6:** Adherence to COVID-19 guidelines over-time: comparison across studies: Means, SDs, and Pearson’s *r* correlation with the other variables measured in Study 1 and Study 2.

	*CVD-M*	Gender^a^	Age	C	LP	DE	TE	PV	NV	RK	FP
Study 1	3.48 (0.87)	−0.22^**^	0.14^**^	0.24^**^	−0.10^**^	−0.22^**^	−0.15^**^	−0.23^**^	−0.40^**^	−0.18^**^	−0.35^**^
Study 2	3.55 (0.94)	−0.23^**^	0.13^**^	0.12^**^	−0.11^*^	−0.16^**^	−0.05	−0.12^*^	−0.35^**^	−0.13^**^	−0.25^**^

* *p* < 0.05;

** *p* < 0.01.

C, Conscientiousness; LP, Lapses; DE, disorganization-errors; TE, temporal-errors; PV, procedural-violations; NV, Noncompliance-Violations; RK, risks; FP, Failure Proneness; and CVD, COVID19 guideline adherence.

a “female” is coded as “0” and “male” is coded as “1.”

Hence, a positive correlation indicates a higher score for men, and a negative correlation indicates a higher score for women.

Noteworthy, in Study 2, the correlations between adherence to the measures and other variables (Conscientiousness and FP) were weaker than in Study 1. A plausible reason for this pattern could be that in Study 2, considerations other than personality came into play and accounted for a portion of the variance in prevention behavior. Study 2 was conducted just as the third COVID-19 wave subsided. At that time, the public was divided in their views on how to balance the health-economics tradeoff, and on where to draw the line between safe and unsafe practices. This variance in attitudes, which was possibly affected by contextual factors such as personal and social preoccupations (Emanuel et al., 2022), could have yielded variance in adherence behavior and diluted the predictive validity of personal characteristics.

### FP, CFQ, and adherence with COVID-19 guidelines

Although FP predicted COVID-19 behavior, CFQ did not. The analysis of the first-order correlation between CFQ and adherence yielded no association. Although this is somewhat different than previous results (Thoma et al., 2021—who found a negative correlation between CFQ and precautionary behavior in the first-order correlations, but not a unique contribution of cognitive failures in the regression model), it is expected in light of the relationships obtained between CFQ and the first order factors of FP. CFQ was strongly correlated to the Lapses factor of FP, but had only a weak correlation with the intentional factors (and in particular Noncompliance-Violations), which predicted COVID-19 behavior (see Table 4).

### Limitations and further research

Our study’s findings should be considered in light of its main methodological limitations, which limit the generalizability of the results:

Firstly, while our sample was large, it should not be considered representative, as most of our participants were young and educated. As compared to adults, young people are less likely to experience severe COVID-19 symptoms. Although they do contribute to the spread of the virus, they are expected to perceive the costs of precautionary behavior as outweighing the benefits. Thus, the conflict between the micro level of the individual and the macro level of a society (which is related to rule violation) might be more salient for them, and consequently they might be more prone to Noncompliance-Violations. In addition, our sample was more educated than the general population. The vast majority of the participants had an academic degree or were undergraduate students who are expected to get an academic degree within 2 years. Although previous research (e.g., Broadbent et al., 1982; Könen and Karbach, 2020) has indicated that behavioral failures (and in particular cognitive failures) do not appear to be very closely related to intelligence, cognitive ability or educational level, it may still be the case that the FP is related to these constructs. Moreover, some authors found that non-compliance, especially with hygiene-related measures, was more prevalent in individuals with higher education (O’Connell et al., 2020; Nivette et al., 2021), while others found that education had no effect on compliance (Oosterhoff et al., 2020). Different types of behavioral failures have different demographic correlates (Lawton and Parker, 1998; de Winter et al., 2015) and individual variations in a range of demographic factors predict compliance with COVID-19 official precautionary guidelines (Bogg and Milad, 2020; Bailey et al., 2021; Emanuel et al., 2022). Factors, such as age, family status, education, and work obligations may affect how people balance their health-economics trade-off, and where they draw the lines concerning what is safe and what is not. It is vital that our results be validated and refined among additional populations as well. In different populations, different patterns of relationships between failure proneness and noncompliance might surface.

Secondly, as with many studies in this research domain, our findings are based entirely on self-reported behaviors and tendencies. Although self-reported adherence to COVID-19 guidelines and objective adherence data correlate fairly strongly (Gollwitzer et al., 2020), there is still a concern than that correlations between the study variables may be due to CMV (Podsakoff et al., 2003), which denotes a range of response biases that might happen when different variables are assessed *via* similar methods. Self-reporting is susceptible to biases, such as social desirability, maintaining positive self-impressions (Leary and Kowalski, 1990; Fisher, 1993) and retrospective memory biases (Kouchaki and Gino, 2016) and other response tendencies that might yield spurious correlations between study variables. Moreover, people may wish to report in a congruent manner on their behaviors or attitudes they exhibit, if they perceive that a study is investigating a link between the two (Helman and Reed, 2015). Although, the statistical analysis (*the Harman’s single factor test*) that we have conducted to address this concern indicated that in both studies no single factor explained the majority of variance in the data, we believe that in order to further understand how failure proneness contributes to noncompliance, future research should also incorporate objective measures, such as travel tracked *via* GPS, or observations of mask usage.

Thirdly, the internal consistency of the DE subscale is lower than desired. This might be due to its length (two items only). The formula for the Cronbach’s alpha is: *α* = K^*^ mean *r*/[1 + (K-1) ^*^ mean *r*]. Thus, two factors influence the magnitude of *α*: *K* (the number of items selected to constitute the scale) and mean *r*. A small number of scale items would violate tau-equivalence and give a lower reliability coefficient. Hence it is common to find quite low Cronbach values (e.g., 0.50) for scales with less than five items. Longer scales give higher alpha values (Hinton et al., 2014). Subsequent research will refine the scale and expose additional categories of failures. For example, it is unclear whether the items of DE should be treated as a subscale or should be divided into two subscales upon addition of items. In future studies, we plan to improve the internal consistencies of the subscale and try to disentangle the unique contribution of each type of failure to non-adherence and other risky behaviors.

## Data availability statement

The raw data supporting the conclusions of this article will be made available by the authors, without undue reservation.

## Ethics statement

The studies involving human participants were reviewed and approved by The Open University of Israel Ethics Committee and the Tel Aviv Yaffo Ethics Committee. The patients/participants provided their written informed consent to participate in this study.

## Author contributions

Both authors listed have made a substantial, direct, and intellectual contribution to the work and approved it for publication.

## Funding

This research was supported by The Open University of Israel’s Research Fund (Grant no. 41355) and the Academic College of Tel Aviv Yaffo’s Internal Grant Fund.

## Conflict of interest

The authors declare that the research was conducted in the absence of any commercial or financial relationships that could be construed as a potential conflict of interest.

## Publisher’s note

All claims expressed in this article are solely those of the authors and do not necessarily represent those of their affiliated organizations, or those of the publisher, the editors and the reviewers. Any product that may be evaluated in this article, or claim that may be made by its manufacturer, is not guaranteed or endorsed by the publisher.
